# Supplementary material for: Clinical significance of non-thyroidal illness syndrome on disease activity and dyslipidemia in patients with SLE
Source: PLoS One. 2020 Apr 16;15(4):e0231622. doi: 10.1371/journal.pone.0231622 (PMC7162454; doi:10.1371/journal.pone.0231622)
Supplement: Attachment — Submitted filename: comments.docx [file pone.0231622.s001.docx]

This study “Clinical significance of non-thyroidal illness syndrome on disease activity and dyslipidemia in patients with SLE” validated the association between FT3 and SLE disease. They discovered that a low FT3 is associated with SLE disease activity, and a low FT3 is an independent risk factor for dyslipidemia in patients with SLE. This study, while confirmative, provided useful information for FT3 in Chinese population. Despite the authors identified FT3 is a risk factor for dyslipidemia in SLE patients, they didn’t provide the direct correlation analysis between TF3 and biomarkers of lipid profiles.

In addition, this review has the following comments:

1. The authors found that FT3 levels in serum are negatively correlated with the levels of UP, FBG, ESR, BUN, SLEDAI, Cr, as well as CPR. However, the correlations are weak, as shown by the values of correlation coefficients (r) in figure 1. Moreover, it’s unknown how NTIS pathogenesis contributes to these correlations. Therefore, analyzing these correlations in patients with NTIS and in patients without NTIS separately may better address this question.

2. The above concern applies also to relationships between the ApoB/ApoA1 and CRP, ESR, Cr, UP, as well as SLEDAI in figure2.

4. The resolution for Figure1 and Figure2 could be improved.

5. The actual number of recruited patients is 223. However, it’s inconsistent in the article.
