# Supplementary material for: Clinical significance of non-thyroidal illness syndrome on disease activity and dyslipidemia in patients with SLE
Source: PLoS One. 2020 Apr 16;15(4):e0231622. doi: 10.1371/journal.pone.0231622 (PMC7162454; doi:10.1371/journal.pone.0231622)
Supplement: Attachment — Submitted filename: comments-Pone.docx [file pone.0231622.s002.docx]

Mar 12, 2020

Dear Editor

Thank you for your letter regarding our manuscript (PONE-D-19-35454) entitled “Clinical significance of non-thyroidal illness syndrome on disease activity and dyslipidemia in patients with SLE” by Xin Zhang, et al. We have addressed the comments (see below) and revised the manuscript according to the comments. We appreciate the constructive criticisms of the reviewers and believe that our revisions have clarified and strengthened the manuscript.

Specifically, we have made the following revisions in response to the reviewers’ comments:

Comments from the editor:

1. This study “Clinical significance of non-thyroidal illness syndrome on disease activity and dyslipidemia in patients with SLE” validated the association between FT3 and SLE disease. They discovered that a low FT3 is associated with SLE disease activity, and a low FT3 is an independent risk factor for dyslipidemia in patients with SLE. This study, while confirmative, provided useful information for FT3 in Chinese population. Despite the authors identified FT3 is a risk factor for dyslipidemia in SLE patients, they didn’t provide the direct correlation analysis between TF3 and biomarkers of lipid profiles.

Reply: Thank you for you critical comments, we revised our manuscript according your advice (see below).

Comments from the reviewers:

1. The authors found that FT3 levels in serum are negatively correlated with the levels of UP, FBG, ESR, BUN, SLEDAI, Cr, as well as CPR. However, the correlations are weak, as shown by the values of correlation coefficients (r) in figure 1. Moreover, it’s unknown how NTIS pathogenesis contributes to these correlations. Therefore, analyzing these correlations in patients with NTIS and in patients without NTIS separately may better address this question.

Reply: Thank you for you critical comments. We analyzed the associations in patients with NTIS and in patients without NTIS separately. The results showed that the FT3 levels were negatively correlated with 24 h UP (*r* = -0.301; *p* < 0.001; Fig. 1A), BUN (*r* = -0.325; *p* < 0.001; Fig. 1B), Cr (*r* = -0.298; *p* < 0.001; Fig. 1C), as well as SLEDAI (*r* = -0.313; *p* < 0.001; Fig. 1F). However, these correlations were not observed in patients without NTIS (Fig. 2A, 2B, 2C and 2F). In addition, FT3 were negatively correlated with CRP in both NTIS patient (*r* = -0.200; *p* < 0.05; Fig. 1D) and euthyroid patients (*r* = -0.254; *p* < 0.05; Fig. 2D), ESR (*r* = -0.27; *p* < 0.05; Fig. 2E) in NTIS patients rather than in euthyroid patients (Fig. 1E). These results indicate that low levels of FT3 are closely related to a higher SLE disease activity mainly in NTIS patients rather than in euthyroid patients.

SLE is a heterogeneous disease and the aetiology is very complicated. The weak but statistically significant correlations found in this study suggests that NTIS maybe one of the factors impacting the disease activity and long-term prognosis of SLE.

2. The above concern applies also to relationships between the ApoB/ApoA1 and CRP, ESR, Cr, UP, as well as SLEDAI in figure2.

Reply: We analyzed the associations in patients with NTIS and in patients without NTIS separately. We found that the ApoB/ApoA1 ratio was significantly associated with SLEDAI (*r* = 0.251; *p* < 0.01; Fig.3E) but not with CRP, ESR, Cr, or 24 h UP (Fig. 3A, 3B, 3C and 3D) in NTIS patients. In euthyroid patients, the ApoB/ApoA1 ratio was significantly associated with CRP (*r* = 0.428; *p* < 0.001; Fig 4A) and ESR (*r* = 0.315; *p* < 0.01; Fig. 4B), but not with Cr, 24 h UP and SLEDAI (Fig. 4C, 4D and 4F). These results indicate that the atherogenic ApoB/ApoA1 ratio are somewhat related to the SLE disease activity in NTIS patients, as well as immunologic inflammation in euthyroid patients.

3. The resolution for Figure1 and Figure2 could be improved.

Reply: We increased the figure resolution to 300 × 300 dpi.

4. The actual number of recruited patients is 223. However, it’s inconsistent in the article.

Reply：Thank you for your careful review, we corrected this error.

In addition, we deleted the data about fasting blood glucose, because it is neither a criteria of dyslipidemia nor a criteria for SLEDAI.

If you have any questions regarding our manuscript, please don’t hesitate to contact me.

Sincerely yours,

Zhiyong Chen, MD, PhD

chainzhiyong@163.com

Nanjing University Medical School.
